# Nutritional assessment of metabolic syndrome patients with hypopituitarism secondary to pituitary adenomas

**DOI:** 10.1186/1758-5996-7-S1-A116

**Published:** 2015-11-11

**Authors:** Renata Elisabeth Rothen, Débora Zeni, Carolina Garcia Soares Leães, Miriam da Costa Oliveira, Fernanda Michielin Busnello, Júlia Fernanda Semmelmann Pereira-Lima

**Affiliations:** 1 UFCSPA, Porto Alegre, Brazil

## Background

Hypopituitarism, a condition in which the pituitary gland does not produce one or more of its hormones, may cause increased visceral fat deposition, dyslipidemia and decreased muscle mass. Metabolic syndrome is highly prevalent in hypopituitarism, being a complex disorder consisting of metabolic abnormalities, such as central obesity, dyslipidemia, hyperglycemia and hypertension.

## Objective

We performed clinical, laboratory and nutrition assessment in patients with hypopituitarism, pituitary adenomas and metabolic syndrome, attending a tertiary clinic in southern Brazil.

## Materials and methods

This was a cross-sectional study of 36 outpatients, aged 20-75 yrs., with metabolic syndrome whose diagnosis was established based on the International Diabetes Federation (IDF) criteria and hypopituitarism, in the presence or after pituitary adenoma treatment. In the anthropometric assessment the body weight, height, body mass index (BMI) and waist circumference were measured. Furthermore, serum lipid levels and fasting glucose were measured. Nutritional assessment included sociodemographic information, history of diseases, use of medications as well as food intake by a 24-h food recall which was evaluated according to the Brazilian guidelines for metabolic syndrome.

## Results

Nineteen women and 17 men were studied, aged 29-73 yrs. (56.9±9.6), 21 of them presented clinically non-functioning pituitary adenoma, 9 prolactinoma, 4 somatotropinoma, and 2 adrenocorticotropinoma. With respect to hypopituitarism, 28 patients presented panhypopituitarism and 8 isolated hormone deficiency. Mean body mass index was 32.9±5.9kg/m2, 67% of subjects were obese and 33% overweight. Waist circumference was increased in 17.6% and high in 82.3% of men, and increased in 10.5% and high 84.4% in of women. Food intake was characterized by low intake of energy and fiber, adequate intake levels of carbohydrate and fat, and high intake of proteins. Also, patients presented increased levels of plasma cholesterol, triglyceride and glucose.

## Conclusions

In this study, nutritional assessment showed that most patients with hypopituitarism and metabolic syndrome presented class I obesity, and high waist circumference. Also, food intake was characterized by energy and fiber intake below recommended values, adequate in carbohydrate and fat content, and high in protein. When faced these findings, some changes in lifestyle are recommended, including adequate diet and regular physical activity.

